# Benzylalkoholmono(poly)hemiformal: MAK-Begründung, Nachtrag

**DOI:** 10.34865/mb1454860kskd11_1ad

**Published:** 2026-03-30

**Authors:** Andrea Hartwig, MAK Commission

**Affiliations:** 1 Institut für Angewandte Biowissenschaften, Abteilung Lebensmittelchemie und Toxikologie, Karlsruher Institut für Technologie (KIT) Adenauerring 20a, Geb. 50.41 76131 Karlsruhe Deutschland; 2 Ständige Senatskommission zur Prüfung gesundheitsschädlicher Arbeitsstoffe, Deutsche Forschungsgemeinschaft, Kennedyallee 40, 53175 Bonn, Deutschland. Weitere Informationen: Ständige Senatskommission zur Prüfung gesundheitsschädlicher Arbeitsstoffe | DFG

**Keywords:** Benzylalkoholmono(poly)hemiformal, Nase, oberer Atemtrakt, Kanzerogenität, Reizwirkung, Keimzellmutagenität, Sensibilisierung, Formaldehydabspalter, Strukturanalogie, Luft, air

## Abstract

The German Senate Commission for the Investigation of Health Hazards of Chemical Compounds in the Work Area (MAK Commission) has re-evaluated benzyl alcohol mono(poly)hemiformal [14548-60-8] with regard to its carcinogenicity and germ cell mutagenicity classification, its ability to be absorbed through the skin, its sensitization potential and whether an occupational exposure limit value (maximum concentration at the workplace, MAK value) can be derived. Relevant studies were identified from a literature search. Benzyl alcohol mono(poly)hemiformal causes irritation of the eyes in rabbits. The substance is a formaldehyde releaser and is expected to undergo rapid hydrolysis in aqueous solution. For this reason, the effects are attributed to the hydrolysis products formaldehyde and benzyl alcohol. There are no studies available that investigated the carcinogenicity, toxicity and genotoxic potential of benzyl alcohol mono(poly)hemiformal in the upper respiratory tract or nose, which are the likely target organs. Formaldehyde was classified in Carcinogen Category 4 because it induces tumours in nasal tissue at concentrations that exceed its detoxification capacity. As a formaldehyde releaser, the substance could likewise be classified in Carcinogen Category 4. However, because it is not possible to derive a MAK value for benzyl alcohol mono(poly)hemiformal, the substance has been assigned to Carcinogen Category 2 with the footnote “Prerequisite for Category 4 in principle fulfilled, but insufficient data available for the establishment of a MAK or BAT value”. As there are no data for the systemic bioavailability of benzyl alcohol mono(poly)hemiformal and the formaldehyde released by hydrolysis in tissues, there is no experimental evidence that the formaldehyde reaches the germ cells. Therefore, benzyl alcohol mono(poly)hemiformal has been classified in Category 3 B for germ cell mutagens. Benzyl alcohol mono(poly)hemiformal induces skin sensitizing effects and the substance has therefore been designated with “Sh”. Skin contact is not expected to contribute significantly to systemic toxicity.

**Table:** 

**MAK-Wert **	**–**
**Spitzenbegrenzung **	**–**
	
**Hautresorption**	**–**
**Sensibilisierende Wirkung (2004)**	**Sh**
**Krebserzeugende Wirkung (2025)**	**Kategorie 2^a)^**
**Fruchtschädigende Wirkung**	**–**
**Keimzellmutagene Wirkung (2025)**	**Kategorie 3 B**
	
**EKA**	**–**
	
Synonyma	für Monohemiformal: Benzylhemiformal Benzylhydroxymethylether Benzyloxymethanol Hydroxybenzyloxymethan
Chemische Bezeichnung (IUPAC-Name)	Phenylmethoxymethanol
CAS-Nr.	14548-60-8
Formel	Strukturformel von Benzylalkoholmono(poly)hemiformal. 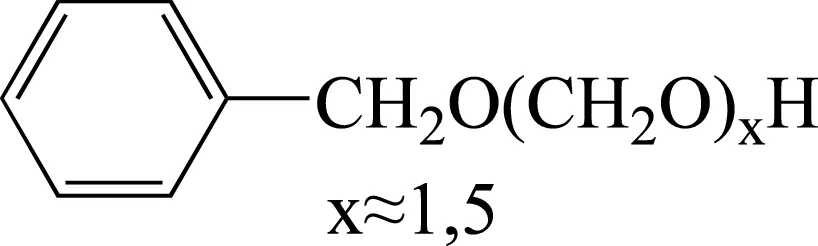
Dampfdruck	0,701 hPa (US EPA 2024)
Siedepunkt	234 °C (US EPA 2024)
Molmasse	138,17 g/mol
Wasserlöslichkeit	25 g/l (Henschler 1989)
log K_OW_	1,3 (ber.; xlogP3) (NCBI 2025)
Herstellung	Addition von Formaldehyd an Benzylalkohol
Hydrolysestabilität	k. A.; bei Kontakt mit Wasser spaltet sich die Substanz zu Formaldehyd und Benzylalkohol

^a)^ Voraussetzung für Kategorie 4 prinzipiell erfüllt, aber Daten für MAK- oder BAT-Wert-Ableitung nicht ausreichend

Hinweis: Formaldehydabspalter. Der Stoff kann gleichzeitig als Dampf und Aerosol vorliegen.

Der Stoff wird unter dem Handelsnamen Preventol^®^D2 vertrieben (nicht zu verwechseln mit Bis(benzyloxy)methan, CAS-Nr. 2749-70-4, mit demselben Handelsnamen) (ECHA 2016). In der Begründung werden der Einfachheit halber die chemische Bezeichnung Benzylalkoholmono(poly)hemiformal und die physikalisch-chemischen Daten zum Monohemiformal (CAS-Nr. 14548-60-8) verwendet, wenn auch der überwiegende Teil der angeführten Untersuchungen mit Preventol^®^D2 durchgeführt worden ist.

Es liegen eine Begründung (Henschler 1989) und ein Nachtrag zur sensibilisierenden Wirkung (Greim 2004) vor. Es erfolgt eine Neubewertung der Daten zur Formaldehydabspaltung nach aktuellem Vorgehen der Kommission (DFG 2025). Unter diesem Aspekt werden in diesem Nachtrag die bewertungsrelevanten Endpunkte reevaluiert.

Benzylalkoholmono(poly)hemiformal ist im Rahmen von REACH vorregistriert (ECHA 2024). Es ist eine Biozid-Registrierung eingereicht, aber bisher sind keine Daten veröffentlicht und es erfolgte keine Zulassung für den Bereich (Kühl)Schmierstoffe (ECHA 2026).

Benzylalkoholmono(poly)hemiformal enthält 26,9 % gebundenen Formaldehyd (Henschler 1989).

## Allgemeiner Wirkungscharakter

Der vermutlich kritische Effekt von Benzylalkoholmono(poly)hemiformal ist die kanzerogene und reizende Wirkung von freigesetztem Formaldehyd am Atemtrakt. Der Stoff wirkt hautsensibilisierend. Für Benzylalkoholmono(poly)hemiformal ergibt sich ein Verdacht auf eine mutagene Wirkung in vitro an Bakterien. Ein Mikronukleustest mit oraler Gabe an Mäusen ist negativ. Daten zur wiederholten inhalativen Exposition, Reproduktionstoxizität, Keimzellmutagenität und Kanzerogenität liegen nicht vor.

## Wirkungsmechanismus

Benzylalkoholmono(poly)hemiformal ist ein Formaldehydabspalter.

## Toxikokinetik und Metabolismus

Es liegen keine Daten mit Benzylalkoholmono(poly)hemiformal vor.

Für eine 0,2%ige nicht mehr reizende Lösung (siehe Abschnitt „Wirkung auf Haut und Schleimhäute“) berechnen sich mit dem Modell von Fiserova-Bergerova et al. (1990) und dem Algorithmus des IH SkinPerm-Modells (Tibaldi et al. 2014) Fluxe von 45 bzw. 5,3 µg/cm^2^ und Stunde. Unter der Annahme einer einstündigen Exposition von 2000 cm^2^ Hautoberfläche würde dies Aufnahmemengen von 90 bzw. 10,6 mg entsprechen.

## Tierexperimentelle Befunde und In-vitro-Untersuchungen

### Wirkung auf Haut und Schleimhäute

Nach einer zweistündigen okklusiven Exposition auf die Haut von Kaninchenohren mit unverdünntem Benzylalkoholmono(poly)hemiformal kam es zu einer leichten, nach acht Stunden zu einer deutlichen Rötung. Nach zwei Tagen traten eine Schwellung und kleine Verätzungsstellen am Applikationsort auf, die bis zum 3. Tag persistierten. Bei einer wässrigen Lösung von 0,2 % war dagegen nach 24-stündiger okklusiver Behandlung der Haut keine Reizwirkung zu erkennen (Henschler 1989).

### Genotoxizität

Benzylalkoholmono(poly)hemiformal war im Mutagenitätstest an dem Salmonella-typhimurium-Stamm TA100 in Anwesenheit eines metabolischem Aktivierungssystems positiv bei einer Konzentration von 400 µg/Platte und über 500 µg/Platte trat Zytotoxizität auf. Die beobachtete mutagene Wirkung der niedrigen Konzentration führte der Studienautor auf den Gehalt an freiem Formaldehyd zurück. In einem Mikronukleustest an männlichen und weiblichen Mäusen, denen zweimal bis zu 500 mg/kg KG oral verabreicht wurden, ließen sich keine Anzeichen einer genotoxischen Wirkung nachweisen (Henschler 1989).

Auch Formaldehyd war am Salmonella-typhimurium-Stamm TA100 in Anwesenheit eines metabolischem Aktivierungssystems positiv (OECD 2002).

Neue Daten liegen zu diesem Endpunkt nicht vor.

## Bewertung 

Das grundlegende Vorgehen zur Bewertung eines Arbeitsstoffes ist der MAK- und BAT-Werte-Liste zu entnehmen (DFG 2025).

Kritische Effekte sind die kanzerogene und lokal reizende Wirkung des Hydrolyseprodukts Formaldehyd.

**Krebserzeugende Wirkung. **Es liegen keine Untersuchungen zur krebserzeugenden Wirkung von Benzylalkoholmono(poly)hemiformal vor. Benzylalkoholmono(poly)hemiformal weist in den vorliegenden Untersuchungen in vivo kein genotoxisches Potenzial auf, eine mögliche genotoxische Wirkung am wahrscheinlichen Zielgewebe des oberen Atemtrakts bzw. der Nase (wie bei Formaldehyd) ist jedoch nicht untersucht.

Aufgrund des Fehlens genauer Daten zur Hydrolysegeschwindigkeit wird als Worst-Case-Abschätzung davon ausgegangen, dass Benzylalkoholmono(poly)hemiformal beim Auftreffen im Atemtrakt sofort vollständig zu Formaldehyd und Benzylalkohol hydrolysiert und der Abbau des aus Benzylalkoholmono(poly)hemiformal entstehenden Formaldehyds langsamer erfolgt als dessen Bildung.

Die lokale Kanzerogenität des Hydrolyseprodukts Formaldehyd ist ausführlich dokumentiert (Greim 2000; Hartwig 2010). Formaldehyd ruft Nasentumoren hervor, jedoch erst bei Konzentrationen, die die Entgiftungskapazitäten des Nasengewebes überschreiten. Daher ist Formaldehyd in Kanzerogenitäts-Kategorie 4 eingestuft.

Aufgrund der lokalen kanzerogenen Wirkung von Formaldehyd könnte Benzylalkoholmono(poly)hemiformal in Analogie zu Formaldehyd in Kanzerogenitäts-Kategorie 4 eingestuft werden. Da jedoch kein MAK-Wert für Benzylalkoholmono(poly)hemiformal abgeleitet werden kann, wird der Stoff der Kanzerogenitäts-Kategorie 2 zugeordnet und erhält die Fußnote „Voraussetzung für Kategorie 4 prinzipiell erfüllt, aber Daten für MAK- oder BAT-Wert-Ableitung nicht ausreichend“.

**MAK-Wert. **Es liegen keine Inhalationsstudien mit Benzylalkoholmono(poly)hemiformal am Menschen oder am Tier vor, aus denen ein MAK-Wert abgeleitet werden kann.

Benzylalkoholmono(poly)hemiformal ist ein Formaldehydabspalter. Formaldehyd zeigt deutliche Reizwirkungen in der Nase und kann bei chronischer Exposition Tumoren im Epithel der Nasenschleimhaut erzeugen. Die Entgiftungskapazitäten des Nasengewebes für Formaldehyd werden bei einem MAK-Wert von 0,3 ml/m^3^ (0,37 mg/m^3^) nicht überschritten (Greim 2000; Hartwig 2010).

Der Dampfdruck (0,701 hPa) und der Siedepunkt (234 °C) von Benzylalkoholmono(poly)hemiformal zeigen, dass der Stoff auch als Dampf-Aerosol-Gemisch vorliegen kann. Für Aerosole ist durch die Impaktierung im Atemtrakt mit einer stärkeren Wirkung als die durch dampfförmigen Formaldehyd verursachte zu rechnen, wie mit einem anderen Formaldehydabspalter, zu dem eine Inhalationsstudie vorliegt, gezeigt wurde (siehe Begründung N,N′,N′′-Tris(β-hydroxyethyl)hexahydro-1,3,5-triazin; Hartwig und MAK Commission 2023).

Für Benzylalkoholmono(poly)hemiformal kann somit kein MAK-Wert festgesetzt werden. Eine Spitzenbegrenzung entfällt.

Bei Anwendung in verdünnten wässrigen Lösungen sollte mit einer vollständigen Hydrolyse gerechnet und daher der MAK-Wert für Formaldehyd (Greim 2000; Hartwig 2010) eingehalten werden.

**Fruchtschädigende Wirkung. **Da kein MAK-Wert abgeleitet wird, entfällt die Zuordnung zu einer risikobasierten Schwangerschaftsgruppe. Da weder valide Daten zur Entwicklungstoxizität noch Hinweise auf eine mögliche entwicklungstoxische Wirkung vorliegen, lässt sich ein entsprechender Verdacht (Gruppe B (Verdacht)) nicht begründen.

**Keimzellmutagene Wirkung. **Aus den vorliegenden Untersuchungen ergibt sich für Benzylalkoholmono(poly)hemiformal kein Verdacht auf eine klastogene Wirkung in vivo in somatischen Zellen. Jedoch wirkt der Stoff, wie Formaldehyd, in S. typhimurium TA100 mutagen (Henschler 1989). Neue Daten zur genotoxischen Wirkung oder Untersuchungen zur Wirkung auf die Keimzellen liegen nicht vor.

Wird davon ausgegangen, dass nach Inhalation durch Hydrolyse sofort der gesamte Formaldehyd im oberen Atemtrakt freigesetzt wird, ist dieser wahrscheinlich nicht systemisch verfügbar. Daten dazu fehlen jedoch.

Theoretisch könnte Benzylalkoholmono(poly)hemiformal in Analogie zu Formaldehyd in die Kategorie 5 für Keimzellmutagene (Greim 2000) eingestuft werden, jedoch kann für Benzylalkoholmono(poly)hemiformal kein MAK-Wert aufgestellt werden.

Da Daten zur systemischen Bioverfügbarkeit von Benzylalkoholmono(poly)hemiformal und dem durch Hydrolyse freigesetzten Formaldehyd fehlen, liegt kein experimenteller Beleg vor, dass der freigesetzte Formaldehyd in aktiver Form die Keimzellen erreicht. Daher wird Benzylalkoholmono(poly)hemiformal in Kategorie 3 B für Keimzellmutagene eingestuft.

**Hautresorption. **Für den Menschen lässt sich aus einer Modellrechnung eine maximale dermale Aufnahme von 90 mg Benzylalkoholmono(poly)hemiformal bei Exposition gegen eine 0,2%ige nicht mehr reizende Lösung unter Standardbedingungen (2000 cm^2^ Hautoberfläche, eine Stunde Exposition) abschätzen. Dies entspricht 24 mg Formaldehyd. Die Annahme, dass Benzylalkoholmono(poly)hemiformal sofort vollständig zu Formaldehyd und Benzylalkohol hydrolysiert ist eine Worst-Case-Betrachtung.

Unter Annahme einer konstanten Resorptionsrate und, im Sinne einer Worst-Case-Betrachtung, raschen Hydrolyse des Benzylalkoholmono(poly)hemiformal werden bei 24 mg Aufnahme in einer Stunde demnach maximal 0,5 mg Formaldehyd/1,25 min (mittlere Halbwertszeit des Formaldehyds im Blut; Kaden et al. 2010) resorbiert bzw. freigesetzt. Die transdermale Aufnahme in den letzten sechs Halbwertszeit-Intervallen führt somit im Fließgleichgewicht zu einer im Blut zirkulierenden Formaldehydmenge von (0,5 + 0,25 + 0,13 + 0,06 + 0,03 + 0,015) mg = 1,5 mg. Der physiologische Formaldehydspiegel im Blut des Menschen beträgt etwa 2–3 mg/l (10–15 mg in 5 l Blut, frei und reversibel gebunden) (Heck et al. 1985).

Dieser zusätzliche Eintrag von Formaldehyd durch transdermal aufgenommenes Benzylalkoholmono(poly)hemiformal erhöht demnach die Gleichgewichtskonzentration von Formaldehyd im Blut unbedeutend, sodass Benzylalkoholmono(poly)hemiformal weiterhin nicht mit „H“ markiert wird.

**Sensibilisierende Wirkung. **Aktuelle Daten zur hautsensibilisierenden Wirkung beim Menschen bestätigen weiterhin die sensibilisierende Wirkung, die sicherlich auch auf die Freisetzung von Formaldehyd zurückgeführt werden kann. Anhand der aktuellen Datenlage gibt es keine Handhabe für eine Änderung der Markierung und Benzylalkoholmono(poly)hemiformal wird weiterhin mit „Sh“ markiert. Zur atemwegssensibilisierenden Wirkung liegen keine Daten vor, sodass auch weiterhin keine Markierung mit „Sa“ erfolgt.
